# A broken link: Knowledge of carbohydrate requirements do not predict carbohydrate intake around competition in endurance athletes

**DOI:** 10.1002/ejsc.12183

**Published:** 2024-08-28

**Authors:** Gemma Sampson, James. P. Morton, José. L. Areta

**Affiliations:** ^1^ Liverpool John Moores University Liverpool UK

**Keywords:** carbohydrate, endurance athlete, nutrition knowledge questionnaire, sports nutrition

## Abstract

Endurance athletes fail to meet carbohydrate (CHO) guidelines for competition, which may be due to limited knowledge. However, the relationship between knowledge and practice in this population is unknown. To investigate this, we assessed the dietary intake in 50 athletes (37 females) who completed endurance events ≥2.5 h in duration and compared CHO intake against the carbohydrates for endurance athletes in competition questionnaire validated nutrition knowledge questionnaire, with specific questions related to CHO loading, pre‐competition meal and during‐competition intake. CHO‐loading guidelines (10–12 g · kg^−1^ · day^−1^) were met in practice by *n* = 5 (10%), but there was no relationship between identified requirements (range 0–12 g · kg^−1^ · day^−1^) and actual intake (*r*_*s*_ = 0.133, *p* = 0.358), with the *n* = 18 (36%) who correctly identified requirements, ingesting 6.1 ± 1.9 g · kg^−1^ · day^−1^. CHO intake for pre‐competition meal guidelines (1–4 g · kg^−1^) was met in practice by *n* = 40 (80%), but there was no relationship between identified requirements (range 0 to >4 g · kg^−1^) and actual intake (*r*_*s*_ = 0.101, *p* = 0.487), with *n* = 19 (38%) who correctly identified guidelines requirements, ingesting 1.4 ± 0.6 g · kg^−1^. CHO intake during‐competition guidelines (60–90 g · h^−1^) was met in practice by *n* = 18 (36%), but there was no relationship between the amounts of CHO required (range 30 to >90 g/h) and actual intake (*r*_*s*_ = 0.028, *p* = 0.849), with *n* = 32 (64%) who correctly identified guidelines requirements, ingesting 56 ± 20 g · h^−1^. Results show no relationship between the knowledge of CHO recommendations and practice, suggesting that theoretical knowledge does not guarantee the achievement of best practice and other important factors may ultimately determine practice.

## Highlights

- The carbohydrates for endurance athletes in competition questionnaire (CEAC‐Q) score did not show any association between theoretical knowledge and actual intake for carbohydrate (CHO) loading, pre‐competition, or during competition scenarios, indicating that athletes do not necessarily apply what they know.
- Despite 30% of athletes meeting the guidelines for CHO loading, and CHO intake during competition, there was no clear relationship between practice and their knowledge of these guidelines as measured by the CEAC‐Q.
- The discrepancy between theoretical knowledge and actual intake within real‐world competition highlights that other factors may be important barriers and facilitators to translate scientific knowledge and bridge the gap into optimal dietary practices of endurance athletes.

## 1 INTRODUCTION

Current carbohydrate (CHO) dietary guidelines are based upon an extensive body of literature which show endurance performance benefits with the optimal CHO intake before and during competition (Burke et al., 2011; Jeukendrup, 2004; Kerksick et al., 2017; Thomas et al., 2016). Nonetheless, a clear mismatch exists between current sports nutrition recommendations and CHO intake of athletes in competition (Heikura et al., 2017; Janse van Rensburg et al., 2018; McLeman et al., 2019). For example, ‘suboptimal’ CHO‐loading intakes of 3.5–7.3 g · kg^−1^ have been shown in amateur cyclists and multisport athletes (Armstrong et al., 2012; Havemann et al., 2008; Masson et al., 2016), which fall short of the 10–12 g · kg^−1^ currently recommended (Thomas et al., 2016). Likewise, CHO intake during competition varies greatly between endurance athletes competing in different sports, distances and athlete level with mean CHO intakes ranging between 12 and 94 g · h^−1^ (Armstrong et al., 2012; Cox et al., 2010; Havemann et al., 2008; Martinez et al., 2018; Muros et al., 2019; Pfeiffer et al., 2012), which are typically lower than the 30–90 g · h^−1^ currently recommended—depending on the duration of the event—(Thomas et al., 2016). The reason for this mismatch is unknown, but endurance athletes have a moderate knowledge of CHO guidelines which may in part explain why many fail to achieve recommended CHO intakes for competition (Black et al., 2012; Heikura et al., 2017; Masson et al., 2016; Spronk et al., 2015). However, the degree to which this mismatch in practice is primarily due to gaps in the knowledge of CHO guidelines or other reasons is currently unknown.

Nutrition knowledge is an essential component driving general dietary behaviour (Spronk et al., 2015), yet little is understood about endurance athletes knowledge of CHO guidelines and how they relate to practice within competition. Lack of knowledge of CHO guidelines may be the primary factor, given runners and triathletes are documented to lack general nutrition knowledge (Doering et al., 2016; McLeman et al., 2019). In support of this, we recently developed a novel questionnaire, the carbohydrates for endurance athletes in competition (CEAC‐Q) (Sampson et al., 2022) and subsequently assessed an international cohort of 1016 endurance athletes (Sampson et al., 2023). Our findings have shown that, on average, endurance athletes have limited knowledge of CHO requirements for competition (50 ± 20% CEAC‐Q score) (Sampson et al., 2023). Under the assumption that knowledge determines dietary practice, athletes who lack sports nutrition knowledge would be less likely to adequately fuel before, during or after training and competition, potentially compromising their performance (Black et al., 2012; Havemann et al., 2008; McLeman et al., 2019; Spronk et al., 2015). While it is logical to assume a strong relationship between theoretical nutrition knowledge and practice, the association between knowledge of current CHO guidelines and dietary habits of athletes in competition is not yet characterised.

Therefore, the primary aim of this study was to determine the association between knowledge and dietary practice of contemporary CHO guidelines in a cohort of endurance athletes in a real‐world competition setting. We hypothesised that athletes correctly identifying the CHO recommendations for competition would show best practice CHO dietary intakes within competition.

## 2 METHODS

### 2.1 Participants

A total of 50 (*n* = 37 female) amateur and professional able‐bodied endurance athletes completed the study (age: 29 ± 7 years, body mass: 61.4 ± 8.6 kg). Athletes had a racing experience of 1–3 years (*n* = 12, 24%), 3–5 years (*n* = 8, 16%), 5–10 years (*n* = 16, 32%) or >10 years (*n* = 14, 28%). Over half (*n* = 29, 58%) of the athletes reported working with a registered sports nutritionist or dietitian. Athletes were defined as professionals if they were full‐time athletes, registered with their relevant international governing body and economically reliant on their athlete profession. Their level could be described as ‘elite athletes/international’ (Tier 4) or ‘World Class’ (Tier 5) according to McKay’s framework for sport science research (McKay et al., 2022). Primary sources of nutrition information were from a registered sports nutritionist dietitian (*n* = 18, 36%), self‐directed learning through websites books or online resources (*n* = 14, 28%), coach (*n* = 5, 10%), other athletes (*n* = 5, 10%), sports scientist (*n* = 4, 8%) or did not seek nutrition advice (*n* = 4, 8%). Participant characteristics are shown in Table 1. Participants were recruited following a convenience sampling methodology. The study was conducted in accordance with the Declaration of Helsinki and was approved by the Ethics Committee Liverpool John Moores University [19/SPS/026]. The participant’s written informed consent was obtained after they were informed of the purpose and procedures of the study.

**TABLE 1:** Participants’ characteristics and CEAC‐Q score by competitive level, gender and sport.

		Competitive level		Gender		Sport		
	All (*n* = 50)	Amateur (*n* = 21)	Professional (*n* = 29)	Male (*n* = 13)	Female (*n* = 37)	Cycling (*n* = 17)	Triathlon (*n* = 29)	Running (*n* = 4)
Age (*y*)	29.2 ± 6.5	26.7 ± 6.1	31.0 ± 6.3	25.5 ± 4.1	30.5 ± 6.8	24.9 ± 5.3	31.3 ± 6.2	32.5 ± 5.3
Body mass (kg)	61.4 ± 8.6	60.7 ± 9.5	61.9 ± 8.0	72.0 ± 6.8	57.6 ± 5.5	59.4 ± 7.5	62.9 ± 9.4	59.0 ± 6.4
CEAC‐Q total score (%)	55 ± 15	55 ± 17	55 ± 13	56 ± 18	54 ± 14	58 ± 14	53 ± 15	48 ± 20

*Note*: There were no significant differences in CEAC‐Q scores between cyclists, triathletes and runners (*p* = 0.363), amateur or professional athletes (*p* = 0.961), male or female athletes (*p* = 0.680).

Abbreviation: CEAC‐Q, carbohydrates for endurance athletes in competition questionnaire.

### 2.2 Events

Participants competed in one of 19 national and international events; road cycling (road race, *n* = 17), triathlon (half ironman and ironman, *n* = 29) and running (marathon, *n* = 4) between September 2019 and January 2020. Endurance events consisted of 1‐day cycling (UCI World Championships Harrogate, Vuelta CV Feminas, L’Etape Australia, 50‐mile TT) and a multi‐stage cycling race (Tour of Tasmania); middle distance triathlon (Western Sydney, Taupo, Indian Wells, Bahrain, Challenge Daytona), Ironman® triathlon (Western Australia, Wales, Lanzarote, Cozumel, Arizona) and marathon running (Frankfurt, Valencia, Malaga).

### 2.3 Overview of research protocol

**FIGURE 1:**
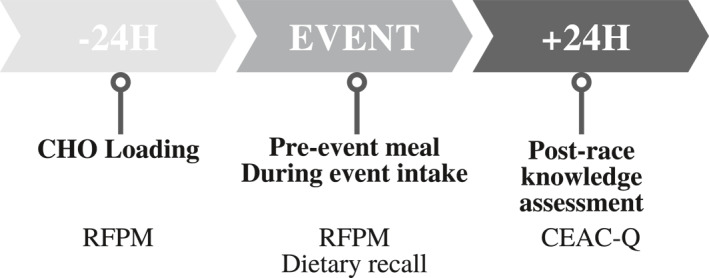
Study timeline around competition period (top) assessment of dietary intake and knowledge (bottom). Dietary intake was assessed using the remote food photography method (RFPM) 24 h in the lead‐up to competition to assess carbohydrate (CHO) loading and pre‐event meal, with the dietary intake during the event assessed with a dietary recall. One day after the event, the knowledge of carbohydrate for competition was assessed using the Carbohydrate for endurance athletes in competition questionnaire (CEAC‐Q). Following recruitment, dietary intake was collected over two consecutive days including the day prior to competition (CHO loading) and competition day for pre‐event meals and intake during the event (which was recalled immediately after the event). Carbohydrate (CHO) knowledge was assessed using Carbohydrate for endurance athletes in competition questionnaire (CEAC‐Q).

This was an observational study conducted in two phases exploring the association between CHO knowledge and practice of endurance athletes during competition through quantitative (current study) and qualitative methods. Knowledge of CHO requirements for competition was assessed using the carbohydrates for endurance athletes in competition (CEAC‐Q) (Sampson et al., 2022) which was completed the day after competition, to mitigate the possibility of the questionnaire influencing behaviour. Dietary intake recorded the period around competition during the 24 h prior to the event and the main meal eaten immediately before the event using the remote food photography methodology (RFPM) (Martin et al., 2012; Stables et al., 2021). Due to the impossibility of using RFPM during competition, individuals provided a dietary recall of what was ingested during the race immediately after finishing their race (Figure 1). In phase 2, participants partook in a semi‐structured interview exploring planned intake, beliefs and reasons for any deviation (manuscript in preparation). No attempt was made to influence or alter race nutrition practices throughout the course of this study. Performance data regarding finishing time for each athlete was published on the event organisers official website and recorded for analysis.

### 2.4 Dietary intake collection and analysis of nutrient intake

Dietary intake was recorded using the remote food photography method (RFPM) (Martin et al., 2009a, 2012; Stables et al., 2021) for all food and fluids consumed in the day before competition (24 h) as time period associated with CHO loading (*CHO‐load*) and for meals before the competitive event (*CHO pre‐competition*; typically breakfast and lunch). Each participant was debriefed on the requirements of this methodology and nudged daily to send food photographs using a smart phone to the lead researcher in real time using WhatsApp Messenger (Facebook, Inc) together with any relevant details of brand labels, cooking methods and description of ingredients (Martin et al., 2009a, 2012). Due to the nature of the events and incapacity to report food intake during competition (*CHO during*), a retrospective food recall was conducted with each participant immediately after the event via smart phone communication. Triathletes and runners reported any cups of fluid collected during the race while running past feed zones. The volumes of cups collected during competition were estimated as 150 mL, which is the maximum volume of fluid likely to be consumed in a real‐life running race (Burke et al., 2005). All dietary supplements and foods consumed were analysed according to manufacturer product labels or website dietary information. Dietary intake was then analysed through Nutritics software (Nutritics, Limited) by the same researcher with 12 years of experience in dietary analysis.

### 2.5 Carbohydrate knowledge and comparison to intake

CHO knowledge was assessed and scored using the CHO for endurance athletes in competition questionnaire (CEAC‐Q) as previously described (Sampson et al., 2022). The CEAC‐Q was administered online using the SurveyMonkey software (https://www.surveymonkey.com) the day following the event so as not to influence dietary choices. We used three specific subsections and questions (Q8, 11, 18c) (Sampson et al., 2022) assessing the knowledge of CHO guidelines that were directly comparable to the collected dietary intake, namely Subsection 2 CHO Loading (9–12 g · kg^−1^), subsection 3 pre‐competition CHO meal (1–4 g · kg^−1^) and Subsection 4 CHO during competition (60–90 g · h^−1^) for events >2.5 h in duration. Actual CHO intake for each time period was compared to the score of each relevant sub‐sections of the CEAC‐Q questionnaire (sections Q8, 11 and 18c) to determine association between intake and knowledge.

### 2.6 Data analysis and statistics

Data were screened for missing values, outliers, normality and skewness. One‐way ANOVA analysed differences between groups in continuous variables and the *X* ^2^ test was applied to compare between groups for categorical variables. Correlations between total CEAC‐Q score, CEAC‐Q subsection scores, specific CHO guideline questions (Q8, 11, 18c) and CHO intake at each time point were analysed using the Pearson correlation coefficient for interval and Spearman’s rank‐order correlation for ordinal data. All statistical analyses were conducted using the IBM SPSS version 26 software (IBM Corp.) with results reported as mean ± SD with a significance level of *p* < 0.05.

## 3 RESULTS

### 3.1 Event characteristics and finishing times

Participants took part in one of 19 separate endurance sports competition events. Mean duration was >150 min for all 19 events; 1‐day (four separate events; *n* = 4; 225 ± 69 min) and multi‐stage cycling (one event; *n* = 1; 169 ± 8 min); 70.3 half Ironman® triathlon (five separate events; *n* = 5; 245 ± 44 min) and full Ironman® triathlon (five separate events; *n* = 5; 552 ± 88 min) and marathon (three separate events; *n* = 4; 174 ± 30).

### 3.2 Carbohydrate loading intake consumed 24 h prior to competition

**FIGURE 2:**
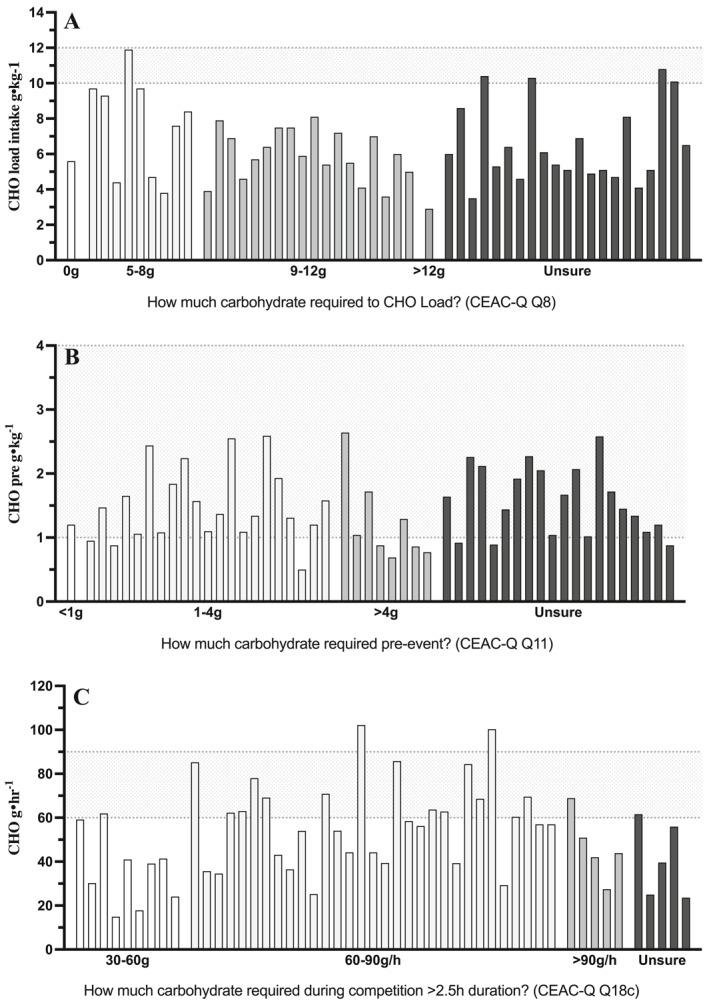
Individual athlete carbohydrate intake categorised by the selected CEAC‐Q target throughout real‐world competition. Graded shading within columns represents different CEAC‐Q answers for the questions C8, C11 and C18c as per values on the *x*‐axis. Each column represents an individual athlete CHO intake compared to recommended intakes for each time point. Dotted lines represent CHO guidelines for each time point whereby (A) CEAC‐Q Q8, CHO load 10–12 g · kg · bm^−1^; (B) CEAC‐Q 11, CHO pre‐competition 1–4 g · kg · bm^−1^; (C) CEAC‐Q Q18c CHO during 60–90 g · h^−1^ ‘0 g’ in A represents the response ‘CHO is never required’.

In the 24‐h period on the day prior to competition, 90% of athletes failed to meet guidelines for CHO loading with mean relative intakes of 6.5 ± 2.2 g CHO.kg (range 2.9–11.9 g · kg^−1^) (Table 2) and CHO intakes of 10–12 g · kg^−1^ were only achieved by 5 athletes (Figure 2A, 10%). Absolute CHO loading intakes range between 185 and 678 g (395 ± 134 g) with wide variation between events and individual athletes (Figure 2A). While professional athletes consumed 105 g more absolute CHO than amateur athletes (439 ± 122 and 334 ± 130 g, respectively, *p* = 0.005), both failed to achieve relative CHO loading recommendations (7.2 ± 2.0 and 5.6 ± 2.0 g · kg^−1^, respectively). Similarly, while male athletes consumed 84 g more CHO than females on average (457 ± 143 and 373 ± 126 g, respectively, *p* = 0.052), there were no differences in CHO intake relative to body mass (6.4 ± 2.2 & 6.5 ± 2.2 g · kg^−1^, respectively, *p* = 0.908) and, on average, neither reached the CHO loading intake recommendations. No difference was observed between athletes who had worked with a nutrition professional or not.

**TABLE 2:** Proportion of athletes achieving carbohydrate intake guidelines around competition periods.

	*CHO intake within competition*		CHO Guideline	Athletes who achieved CHO intake guideline, *n* (%)
	(g)	g · kg^−1^ (g · h^−1^)		
CHO loading	395 ± 134	6.5 ± 2.2	10–12 g · kg^−1^	5 (10)
CHO pre‐event meal	91 ± 37	1.5 ± 0.6	1–4 g · kg^−1^	40 (80)
CHO during competition	289 ± 226	(52 ± 21)	60–90 g · h^−1^	18 (36)

*Note*: Mean total and relative CHO intakes in relation to CHO loading 24 h prior to competition, CHO consumed in the pre‐event meal or CHO consumed during competition and the proportion of athletes achieving CHO guidelines.

### 3.3 Pre‐competition meal carbohydrate intakes

The mean pre‐competition meal CHO consumed by athletes contained 91 ± 37 g CHO with relative intakes of 1.5 ± 0.6 g CHO.kg^−1^ (Table 2). While most athletes (*n* = 40, 80%) consumed CHO intakes within recommendations (1–4 g · kg^−1^), 10 athletes (20%) consumed <1 g · kg^−1^ in the meal before their event. CHO pre‐competition intakes showed a wide range between individual athletes from 34 to 197 g (0.5–2.6 g · kg^−1^) (Figure 2B). Male athletes consumed 15 g more pre‐event CHO than females on average (102 ± 47 and 87 ± 33 g, respectively, *p* = 0.22), but there were no differences in CHO intake relative to body mass (1.4 ± 0.7 & 1.5 ± 0.5 g · kg^−1^, respectively, *p* = 0.64).

### 3.4 During competition carbohydrate intakes

CHO intake during competition showed a wide range from 15 to 102 g · h^−1^ (Figure 2C). Mean CHO intake during competition was 52 g ± 20 g · h^−1^ with just 18 athletes (32%) consuming the recommended CHO for events lasting >2.5 h in duration (60–90 g · h^−1^) and only 2 consuming >90 g · h^−1^ (Table 3). Suboptimal CHO intakes during competition were observed in 64% athletes, with eight athletes (16%) consuming very low CHO intakes <30 g · h^−1^. Marathon runners consumed considerably less CHO per hour (*n* = 4; 32 ± 10 g · h^−1^) than cyclists (*n* = 17; 48 ± 23 g · h^−1^) or triathletes (*n* = 29; 55 ± 19 g · h^−1^, *p* = 0.005). Professional athletes (*n* = 29) consumed more CHO per hour during competition than amateur athletes (*n* = 21; 61 ± 19 vs. 40 ± 15 g · h^−1^, respectively, *p* < 0.001). Of the 18 athletes who achieved CHO guidelines during competition (Table 2), professional athletes (*n* = 12, 48%) were twice as likely as amateur athletes (*n* = 4, 19%) to achieve CHO intake guidelines during competition >2.5 h. While male athletes consumed 111 g less total CHO during competition than females on average (207 ± 141 & 318 ± 244 g, respectively, *p* = 0.129), there were no differences in relative CHO intake consumed per hour (52 ± 22 & 52 ± 20 g · h^−1^, respectively, *p* = 0.962). No difference was observed between athletes who had worked with a nutrition professional or not.

**TABLE 3:** Knowledge of carbohydrate intake guidelines, actual intake within competition and correlation.

Knowledge of CHO Guidelines (CEAC‐Q)			Actual CHO intake at relevant time‐point		Correlation	
CEAC‐Q question	CEAC‐Q guideline intake responses	*N* correct answers (%)	Total (g)	g · kg^−1^ (Q8 & Q11), g · h ^−1^(Q18c)	Spearmans coefficient (*r*_*s*_)	*p*
**CHO loading (Q8)** When carbohydrate loading before competition, the recommended range of carbohydrate intake per day is?	5–8 g · kg^−1^	7 (14)	428 ± 157	6.6 ± 2.5	0.133	0.358
	**9–12 g** · **kg** ^**−**^ **^1^** **^a^**	18 (36)	389 ± 142	6.1 ± 1.9		
	>12 g · kg^−1^	2 (4)	339 ± 108	5.5 ± 0.1		
	CHO loading never required	2 (4)	312 ± 56	6.1 ± 1.3		
	Unsure	21 (42)	403 ± 132	6.9 ± 2.4		
**CHO pre‐race meal (Q11)** How much carbohydrate should a meal eaten before competition contain?	<1 g · kg^−1^	1 (2)	46	0.8	0.101	0.487
	**1–4 g** · **kg** ^**−**^ **^1^** **^a^**	19 (38)	87 ± 37	1.4 ± 0.6		
	>4 g · kg^−1^	9 (18)	115 ± 46	1.7 ± 0.6		
	Unsure	21 (42)	87 ± 30	1.5 ± 0.5		
**CHO during (Q18c)** How much carbohydrate is recommended per hour during competition lasting >2.5 h duration:	30–60 g · h^−1^	9 (18)	226 ± 173	(53 ± 22)	0.028	0.849
	**60–90 g** · **h** ^**−**^ **^1^** **^a^**	32 (64)	341 ± 245	(56 ± 20)		
	>90 g · h^−1^	4 (8)	150 ± 126	(32 ± 9)		
	Unsure	5 (10)	183 ± 162	(41 ± 15)		

*Note*: Athlete reported knowledge of CHO guidelines intakes for each time point obtained from responses to CEAC‐Q questions Q8: CHO Loading; Q11: CHO pre‐competition; 18c: CHO during >2.5 h duration; and subsequent CHO intake. Bold refers to ‘correct answer’.

^a^ CHO Guideline. Additional response options available for each question, including ‘none’ and ‘CHO is never required’ were not selected by any athletes and are subsequently not represented within the table.

### 3.5 Knowledge of carbohydrate intake guidelines (CEAC‐Q scores)

Athlete’s mean total CEAC‐Q score was 55 ± 15% (range 23%–86%), out of a maximum 100 points. Knowledge levels CEAC‐Q scores of 70%–100% were classified as ‘high’ (*n* = 9, 18%), scores of 40%–69% as ‘moderate’ (*n* = 30, 60%) and scores of 0%–39% as ‘low’ (*n* = 11, 22%), following a similar pattern of frequency as identified in a large international population of athletes (Sampson et al., 2023). No difference was observed between athletes who had not worked with a dietitian and those who had, with respective CEAC‐Q scores of 53 ± 16 and 56 ± 15% (*p* = 0.419). No differences were observed in mean scores between the subsection, each worth a maximum 20 points: Subsection 2: CHO Loading (11 ± 5 points), Subsection 3: CHO Pre‐event (12 ± 4 points) and Subsection 4: CHO during (11 ± 5 points). Further exploration highlighted low theoretical knowledge of CHO loading and pre‐competition guidelines. Only a third of athletes were able to correctly identify the CHO loading intake question of 9–12 g · kg^−1^ (Table 3, Q8; *n* = 18, 36%) and that a pre‐competition meal should contain 1–4 g · kg^−1^ CHO (Table 3, Q11; *n* = 19, 38%). Whereas CHO requirements intake *in‐competition* (60–90 g · h^−1^) were correctly identified by over two thirds of the athletes (*n* = 32, 64%) (Table 3).

### 3.6 No relationship between carbohydrate knowledge and practice

Despite CHO loading, pre‐competition meal and during competition intake guidelines were achieved by 16%, 80% and 32% of individuals, respectively, no relationship was observed between knowledge of guidelines for CHO loading, pre‐event meal or during competition and corresponding CHO intake of athletes (Table 3, Figure 2). The total CEAC‐Q score was not associated with the CHO intake of athletes for CHO loading (*p* = 0.677), CHO pre‐competition (*p* = 0.0563) or CHO during competition (*p* = 0.88). Neither did any correlation exist between CHO intake 24 h prior to competition and subsection 2 score (CHO loading; *p* = 0.28); CHO intake in the meal before competition and subsection 3 score (pre‐competition meal; *p* = 0.85) or CHO intake during event and CEAC‐Q subsection 4 score (CHO during competition; *p* = 0.06). The theoretical knowledge of CHO‐loading requirements (10–12 g · kg^−1^) was correctly identified by *n* = 18 (36%), but they ingested 5.5 ± 0.1 g · kg^−1^ and identified that the amount of CHO was unrelated to the actual intake (*r*_*s*_ = 0.133, *p* = 0.358). CHO intake guidelines for the pre‐competition meal (1–4 g · kg^−1^) were correctly identified by 19 athletes (38%), but the identified amount of CHO required was unrelated to the actual intake, ingesting 1.4 ± 0.6 g · kg^−1^ (*r*_*s*_ = 0.101, *p* = 0.487). CHO intake during‐competition requirements (60–90 g · h^−1^) was correctly identified by 32 individuals (64%), but they ingested 56 ± 20 g · h^−1^ and identified amounts of CHO required was also unrelated to the actual intake (*r*_*s*_ = 0.028, *p* = 0.849).

## 4 DISCUSSION

The main finding of this study is that there was no relationship between knowledge on CHO intake recommendations for competition and dietary practices of endurance athletes around competition. To the best of our knowledge, this is the first study determining the link between theoretical knowledge and practice in athletes in relation to CHO intake for competition. While we observed that some endurance athletes could identify current CHO guidelines, this knowledge is not necessarily reflected in practice. Despite theoretical knowledge typically being considered as a fundamental factor for best practice in nutrition (Heaney et al., 2011), our findings suggest that other barriers and facilitating factors may be as important to translate scientific knowledge into optimal dietary practices of endurance athletes in competition.

Our study was conducted in a highly ecologically valid setting, working with endurance athletes as relevant end‐users (Table 1) competing in real‐world national and international level competitions, using validated tools for the assessment of knowledge and dietary intake such as the recently developed CHO for endurance athletes in competition questionnaire (CEAC‐Q) (Sampson et al., 2022, 2023), and the remote food photography method for dietary intake (Stables et al., 2021). Assessing dietary intake in conjunction with the CEAC‐Q unravels a new frontier in the link between knowledge and practice, whereby gaps in theoretical knowledge and dietary practice of CHO loading and intake during competition clearly exist within endurance athletes.

While CHO guidelines are clear, concise and supported by ample research, available literature shows a clear mismatch between these consensus guidelines and current practice. The reasons for this have not been previously explored, and in the current study, we systematically compared knowledge against actual practices to investigate if knowledge is a key contributing factor. Our findings in relation to CHO intake are comparable to previously reported values in the literature showing a typical intake of endurance athletes prior to competition to be 3.3–5.8 g · kg^−1^ (Armstrong et al., 2012; Atkinson et al., 2011; Havemann et al., 2008; Masson et al., 2016; McLeman et al., 2019; Pugh et al., 2018; Wardenaar et al., 2019) for CHO‐loading and 12–94 g · h^−1^ (Armstrong et al., 2012; Atkinson et al., 2011; Havemann et al., 2008; Hoogervorst et al., 2019; Muros et al., 2019; Saris et al., 1983) for CHO during competition (Table 2). As a caveat, it is worth noting that given dietary assessment in athletes has been shown to result in systematic under reporting of ∼19% (Capling et al., 2017), and with the RFPM between 9% and 13% (Dahl Lassen et al., 2010; Kikunaga et al., 2007; Martin et al., 2009b), there is a possibility that our data inflates the frequency with which our subjects fail to achieve the guidelines. However, applying an inflated 20% adjustment factor and re‐analysing our data only see small increases in the amount of individuals successfully achieving CHO guidelines for CHO‐loading (from 10% to 20%), meal pre‐event (from 80% to 94%) and during exercise (from 36% to 40%). The CEAC‐Q identified a broad range of CHO knowledge in athletes, that was ‘moderate’ (55 ± 15%) on average (Table 1), and comparable to an athlete population‐wide assessment of knowledge using the CEAC‐Q (Sampson et al., 2023). Importantly, however, being able to identify CHO guidelines was unrelated to dietary practice at any time point throughout competition (Table 3). Moreover, there was also no relationship between identifying more carbohydrates as necessary and actual CHO intake (Table 2). Overall, these results indicate that there is no direct link between knowledge and practice as the identified amount of CHO required was unrelated to the actual intake for the three time points (Table 3).

The mismatch between knowledge and practice may in part be explained by a lack of practical skills to plan, adhere to and consume adequate CHO at each time point. This is evidenced in our findings of no relationship between knowing the guidelines (as evidenced by the CEAC‐Q questionnaire) and consuming the identified amount of CHO required in real‐world competition (Table 3). This is similar to previous findings showing no difference between the CHO intake of cyclists who reported to CHO load and those who did not (6.0 vs. 5.6 g · kg^−1^) (Havemann et al., 2008) or research showing that cyclists who reported prior intent to CHO load failed to consume enough CHO in line with best practice (Atkinson et al., 2011). Slightly higher CHO intake in professional versus amateur athletes during exercise in our study may suggest that support from a registered sports nutritionist or dietitian and experience (including various skills and a plan) rather than knowledge per se could be an important contributing factor. Additionally, beliefs and intention regarding the need for CHO intake may play a stronger influencing role on intake than knowledge and may explain why athletes consume less CHO than they know is required. Indeed, 52% of 2550 amateur cyclists reported they did not intend to CHO load prior to their 94.7 km cycling road race because they believed they did not need it, feared gaining weight or had experienced negative gastrointestinal issues (Janse van Rensburg et al., 2018). Sociocultural aspects may influence behaviour, and given that 37/50 participants (74%) in our study were females, who in some populations have shown to display ‘fear of carbohydrates’ (McHaffie et al., 2022), this may have been a contributing factor influencing a lower intake. Surprisingly, we did not observe any differences in CHO intakes within competition between male or female athletes. While nutrition knowledge may be a facilitator, it seems evident that actual dietary choices within competition are strongly influenced by factors other than theoretical knowledge and further understanding regarding deviations from planned intake is required.

These results suggest a broken link between knowledge, and practice exists within endurance athletes, which may be a general feature of sports nutritional practices. Studies exploring the complex link between general nutrition knowledge and dietary behaviour show that many athletes do not apply what they know and that increased nutrition knowledge may not translate to superior dietary changes (Heaney et al., 2008; Heikura et al., 2017; Spronk et al., 2015). When exploring the extent to which factors influence practice beyond nutritional knowledge, the COM‐B model of behaviour change suggests that an athlete must have the capability, opportunity and motivation to change their behaviour (Bentley et al., 2021) and must be ready for, want to and able to make that change (Bartlett et al., 2021). Thus, to consume CHO in alignment with guidelines, athletes must have the knowledge and skills (capability), the social and physical opportunity and motivation to consume that CHO in the presence of competing behaviours (Michie et al., 2011). The broken link between knowledge (capability) and dietary practice (behaviour) may be explained by a range of factors associated to individual capabilities or opportunities including—but not limited to—practical skills, physiological limitations (e.g., gastrointestinal distress), logistical issues and personal beliefs (motivation) about CHO (Heaney et al., 2008, 2011; Pelly et al., 2021). Therefore, nutrition interventions for endurance athletes may need to explore and consider a broader range of facilitators and barriers beyond simply improving the knowledge of CHO guidelines to impact positive changes in dietary behaviour within competition and repair this broken link.

## 5 CONCLUSIONS

In conclusion, our findings indicate the existence of a broken link between the knowledge of CHO guidelines for competition and practice. The reasons for the mismatch remain elusive and highlight the need for qualitative studies investigating the rationale of athletes to explain the mismatch. This may result in new insights to improve practice in athletes and identify capability, opportunity and motivational needs of athletes to optimise nutritional behaviour.

## AUTHOR CONTRIBUTIONS

Study design; José. L. Areta, Gemma Sampson and James. P. Morton. Data analysis; José. L. Areta and Gemma Sampson. Figures; José. L. Areta and Gemma Sampson. Manuscript draft; José. L. Areta and Gemma Sampson. All authors read and approved the final version of the manuscript.

## CONFLICT OF INTEREST STATEMENT

JM is a consultant for Science in Sport (plc). His previous research on CHO metabolism during exercise has been funded by Science in Sport, GlaxoSmithKline and Lucozade Ribena Suntory. GS and Jose Lisandro Areta report no conflict of interest.
